# Values of Stakeholders Involved in Applying Surveillance Technology for People With Dementia in Nursing Homes: Scoping Review

**DOI:** 10.2196/64074

**Published:** 2025-03-20

**Authors:** Daniëlle van Gaans-Riteco, Annerieke Stoop, Eveline Wouters

**Affiliations:** 1 Academic Collaborative Center Care for Older Adults Tranzo, Scientific Center for Care and Wellbeing Tilburg School of Social and Behavioral Sciences, Tilburg University Tilburg The Netherlands; 2 Groenhuysen Roosendaal The Netherlands; 3 School for Allied Health Professions Fontys University of Applied Science Eindhoven The Netherlands

**Keywords:** surveillance technology, nursing home, stakeholders, values, dementia, safety

## Abstract

**Background:**

Due to the progressive nature of dementia, concerns about the safety of nursing home residents are frequently raised. Surveillance technology, enabling visual and auditory monitoring, is often seen as a solution for ensuring safe and efficient care. However, tailoring surveillance technology to individual needs is challenging due to the complex and dynamic care environment involving multiple formal and informal stakeholders, each with unique perspectives.

**Objective:**

This study aims to explore the scientific literature on the perspectives and values of stakeholders involved in applying surveillance technology for people with dementia in nursing homes.

**Methods:**

We conducted a scoping review and systematically searched 5 scientific databases. We identified 31 articles published between 2005 and 2024. Stakeholder characteristics were extracted and synthesized according to the theory of basic human values by Schwartz.

**Results:**

In total, 12 stakeholder groups were identified, with nursing staff, residents, and informal caregivers being the most frequently mentioned. Among stakeholder groups close to residents, values related to benevolence, security, conformity, and tradition were most commonly addressed. Furthermore, values such as self-direction, power, and achievement seemed important to most stakeholder groups.

**Conclusions:**

Several stakeholder groups emphasized the importance of being and feeling involved in the application of surveillance technologies. In addition, they acknowledged the necessity of paying attention to stakeholders’ perspectives and values. Across these stakeholder groups, values related to benevolence, security, and self-direction were represented, although various stakeholders assigned different meanings to these values. Awareness of stakeholders’ perspectives demands a willingness to acknowledge each other’s values and bridge differences.

## Introduction

### Background

Globally, people are living longer. Every country in the world expects growth in the number and proportion of older persons [1]. As the population ages, diseases such as dementia are diagnosed more often because age is their strongest known risk factor [2,3]. Dementia is a major cause of disability and dependency, affecting cognitive abilities and behavior, leading to an inability to signal when help is needed, which is associated with safety concerns [3,4]. Compared with 2015, the number of people affected with dementia will triple by 2050 [3].

In several countries worldwide, the number of care professionals is insufficient to meet the growing care demands [5]. In addition, global shortages of skilled care professionals will increase due to the aging workforce [6,7]. To meet the increasing care demands, documents from the Netherlands government show that by 2024, 1 in 4 employees should work in the health care sector, a figure that should rise to 1 in 3 by 2060 [8,9]. One of the proposed solutions to this challenge is allowing people with dementia to live at home as long as possible. However, due to their increasing care demands, a need for long-term care (LTC) facilities providing high-quality intensive dementia care will continue. Studies in different countries worldwide indicate that approximately 30% to 40% of people with dementia will eventually need a care environment in nursing homes [10-12]. The Netherlands is known for its high percentage of residents receiving end-of-life care in nursing homes, which makes nursing homes the most frequent place of death [13]. Consequently, there has been a large increase in health care expenditures for the population with dementia, especially in LTC facilities [12].

One recommendation to address these societal challenges in home care and nursing homes is to foster investment in health technologies that contribute to sustainable and high-quality care for people with dementia, such as assistive and innovative care technologies [3,14,15]. Care technologies can delay or replace admission in a nursing home and reduce the workload of nursing staff and informal caregivers in community care and nursing homes [16,17]. There are different types of care technologies. One is surveillance technology, which allows visual and auditory monitoring and registration of events, including residents’ activities. Surveillance technologies include tagging and tracking technology, sensors, and audio and video surveillance [18,19]. Surveillance technologies are increasingly focused on supporting autonomy and respecting privacy while enhancing safety and individualized care for people with dementia [7,20-23].

Surveillance technologies are often regarded as a solution for ensuring safe and efficient health care, including in nursing homes [14,19,20]. These technologies have the potential to provide high-quality care and relieve nursing staff as staff shortages increase [7]. Due to the potential benefits of using surveillance technologies for quality of life and care, general attitudes toward these technologies have become more positive [16]. Nonetheless, surveillance technologies can affect privacy, autonomy, and freedom of movement [16,20,21]. Therefore, the use of these technologies should comply with regulations governing privacy and involuntary care, including requirements for subsidiarity, proportionality, and expediency. In addition, the use of surveillance technologies has to be justified in the care plan [24-26].

The application of surveillance technologies for people with dementia living in nursing homes is complex in practice. Successfully implementing care technologies, including surveillance technologies in psychogeriatric nursing homes, appears to be challenging as it involves more than just implementing a technological application successfully used elsewhere [27]. One of the greatest challenges in implementing care technologies such as surveillance technology seems to be integrating technology into the care process. Surveillance technologies affect residents and other primary stakeholders, such as residents’ representatives and formal caregivers [16,28]. The involvement of these primary stakeholders, also known as end users, and secondary stakeholders such as managers, information and communication technology (ICT) employees, developers, and vendors of surveillance technologies is necessary to increase stakeholder commitment [28].

Early involvement of relevant stakeholders increases the likelihood of successful implementation [29]. A prerequisite for their involvement is knowing and acknowledging stakeholders’ cultures, perspectives, and interests [29]. Given the broad spectrum of stakeholders involved in applying surveillance technologies for people with dementia in nursing homes, there is a great diversity of backgrounds, resulting in differences in values and interests [27,30]. Values represent what is (most) important to people and direct their attitudes, behaviors, and actions [31,32]. Differences in stakeholder values and interests can complicate the creation of support among stakeholders [30]. In addition, dealing with different perceptions and values among a range of stakeholders is a major challenge, further exacerbated by a limited understanding of stakeholders’ values [33]. Therefore, a knowledge of these values can help explain decision-making processes, attitudes, and behaviors of persons or groups in different contexts [31,34,35].

This situation necessitates exploring the stakeholder groups that are involved in implementing surveillance technologies, and their respective values. Nursing staff and informal caregivers’ attitudes toward using surveillance technologies [18,36] and their ethical dilemmas when using surveillance technology in psychogeriatric nursing homes have been explored [23,36-38]. For example, Rostad and Stokke [39] noted the high complexity of the LTC setting, involving “wicked problems,” such as many and changing stakeholders, competing interests, and disagreements regarding the nature of problems. However, it remains unclear what these competing interests consist of. In addition, little is known about the variation in the perspectives and values of the stakeholders.

### This Study

To the best of our knowledge, no literature review has been conducted to explore the stakeholders involved, their perspectives, and values in the application of surveillance technologies for people with dementia living in nursing homes. Therefore, this scoping review aimed to explore which stakeholders are described in the scientific literature concerning surveillance technologies for people with dementia in nursing homes. In addition, we seek to identify what is known about these stakeholders’ values.

## Methods

### Overview

We conducted a scoping review to systematically explore, map, and synthesize the characteristics of stakeholders involved in applying surveillance technology for people with dementia in nursing homes and identify existing knowledge gaps. The PRISMA-ScR (Preferred Reporting Items for Systematic Reviews and Meta-Analyses Extension for Scoping Reviews) checklist was used as a guideline for this review [40] (Multimedia Appendix 1). The corresponding steps were followed [41]: (1) identifying research questions; (2) identifying relevant literature in databases; (3) selecting the literature; (4) charting the data; and (5) collating, summarizing, and reporting the results.

### Identifying the Research Questions

The research questions formulated were as follows: (1) Which stakeholders are involved in applying surveillance technology for people with dementia residing in psychogeriatric wards in nursing homes? and (2) What is known about the values of these stakeholders?

### Identifying Relevant Literature

We believed that articles of interest had been published in psychological, health care, medical, nursing, and technological journals. Therefore, we conducted a literature search using the following databases: MEDLINE, CINAHL, PsycINFO, ACM Digital Library, and IEEE Xplore. Search terms encompassed the LTC setting and the use of surveillance technology for people with dementia. A search string for each database was developed and programmed with the help of an information specialist (Multimedia Appendix 2). A search was first performed in April 2022 and fully updated in August 2023 and December 2024.

Studies were eligible for inclusion if surveillance technology was applied to people with dementia in nursing homes or a comparable 24×7 LTC setting. The use of surveillance technology was evaluated or monitored using qualitative, quantitative, or mixed methods designs. Furthermore, studies had to be peer reviewed, written in English or Dutch, and published between 2002 and 2023. The year 2002 was chosen because it was then that literature on the implementation of surveillance technologies became increasingly prevalent. In addition, studies had to mention the stakeholders who were involved in the process of applying surveillance technology. Studies mostly focusing on assistive technologies, such as automatic lights, or supportive technologies, for example, medication dispensers, health care apps for managing chronic diseases, etc were excluded. Studies conducted in an experimental or laboratory setting and nonoriginal research, such as scoping reviews and systematic reviews, were excluded.

### Literature Selection

First, duplicate studies were removed. Title screening was performed by one of the authors (DvG-R), and in case of doubt, one of the other authors (AS) was consulted. Two authors (DvG-R and AS) independently screened abstracts using the literature review management tool Rayyan (Rayyan Systems Inc) [42]. The full text of articles considered eligible by both authors was reviewed for relevance. In all the selection steps, the results were compared and discussed until a consensus was reached. In case of doubt, the third author (EW) was consulted.

### Charting the Data

A format for further data extraction was agreed upon and included the title, authors, year, country, aim, study design, method of data collection, study population, sample size, setting, technology type, an overview of the results per identified stakeholder, and limitations for this scoping review. Using this format, 2 authors (DvG-R and AS) independently reviewed 10 (32%) of the 31 included articles. When the reviews were compared, only minor differences were found. The remaining articles were reviewed by DvG-R, who consulted one of the two other authors (AS or EW) when appropriate. When no consensus about data extraction was reached, the other author (EW or AS) was consulted.

### Collating, Summarizing, and Reporting the Results

We categorized the findings from each article per stakeholder group in data extraction forms. Subsequently, an overview of results per stakeholder group was compiled. Through an inductive process, we categorized our findings into frequently mentioned words, such as acceptance; privacy; safety; freedom of movement; person-centered care; quality of life; quality-of-care characteristics; technology characteristics; and resident characteristics, involvement, concerns, and their values. The findings were linked to human values to deepen an understanding of stakeholders’ perspectives. Describing and defining values is considered complex, and analyzing them is an even greater challenge [43]. Therefore, we used the theory of basic human values by Schwartz et al [31], an empirically tested framework of values that is recognized across many cultures [44]. This theory is an important and well-known theory and is widely used to predict attitudes and behaviors in different contexts and situations [34,35]. These values are grounded in the 3 universal requirements of human existence: the needs of individuals as biological organisms, requirements of coordinated social interaction, and survival and welfare needs of groups [31]. This framework conceptualizes values ordered by importance relative to one another, and they form a system of priorities for groups, societies, and individuals [45]. The refined theory of basic human values has 19 values grouped into 4 higher-order categories as follows: openness to change, self-enhancement, conservation, and self-transcendence [31]. Table 1 presents the motivational goals of the Schwartz values based on the circular motivational continuum [31].

**Table 1:** The 19 values of Schwartz et al [31] explained in terms of their motivational goals based on the circular motivational continuum.

Higher order value and values			Conceptual definition in terms of motivational goals
**Openness to changes**			
	Self-direction-thought	Freedom to cultivate one’s own ideas and abilities	
	Self-direction-action	Freedom to determine one’s own actions	
	Stimulation	Excitement, novelty, and change	
**Self-enhancement**			
	Hedonism	Pleasure and sensuous gratification	
	Achievement	Success according to social standards	
	Power-dominance	Power through exercising control over people	
	Power-resources	Power to control material and social resources	
**Conservation**			
	Face	Security and power through maintaining one’s public image and avoiding humiliation	
	Security-personal	Safety in one’s immediate environment	
	Security-societal	Safety and stability in the wider society	
	Tradition	Maintaining and preserving cultural, family, or religious traditions	
	Conformity-rules	Compliance with rules, laws, and formal obligations	
	Conformity-interpersonal	Avoidance of upsetting or harming other people	
**Self-transcendence**			
	Humility	Recognizing one’s insignificance in the larger scheme of things	
	Benevolence-dependability	Being a reliable and trustworthy member of the ingroup	
	Benevolence-caring	Devotion to the welfare and well-being of ingroup members and being empathic	
	Universalism-concern	Commitment to equality, justice, and protection for all people	
	Universalism-nature	Preservation of the natural environment	
	Universalism-tolerance	Acceptance and understanding of those who are different from oneself	

## Results

### General Findings

**Figure 1:**
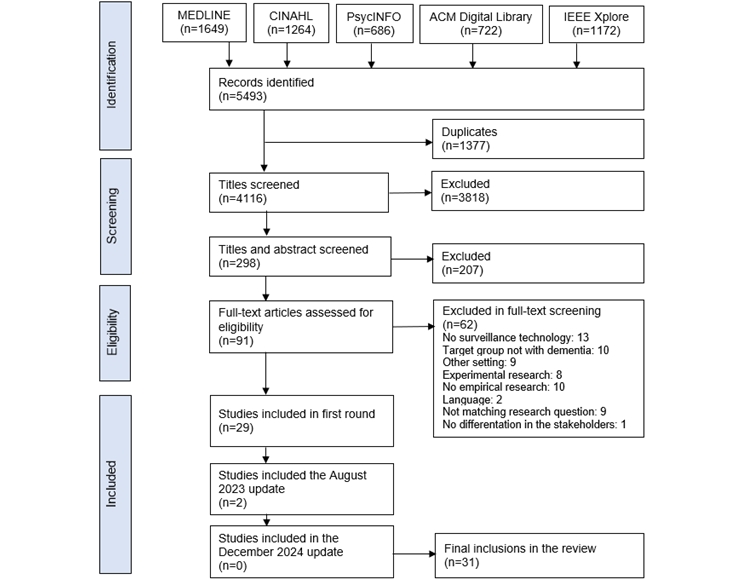
Flowchart of the selection process.

We identified 4116 unique studies. After screening titles, 298 (7.2%) abstracts were screened. After applying inclusion and exclusion criteria, we selected 91 (30.5%) articles for full-text screening. Eventually, we included 29 (32%) articles in this study. The search was updated in August 2023, and December 2024, resulting in 2 additional articles, bringing the total to 31 articles. The included studies were published between 2005 and 2023. Studies were conducted in 14 countries: The Netherlands (n=7, 23%), Finland (n=2, 6%), Denmark (n=1, 3%), Norway (n=2, 6%), Sweden (n=2, 6%), Spain (n=1, 3%), Germany (n=1, 3%), Switzerland (n=1, 3%), the United Kingdom (n=3, 10%), the United States (n=5, 16%), Canada (n=3, 10%), Mexico (n=1, 3%), Singapore (n=1, 3%), and China (n=1, 3%). A total of 10 (32%) articles had a quantitative research design, 18 (58%) articles had a qualitative design, and 3 (10%) articles had a mixed methods design. Figure 1 shows the flowchart of the selection process. The key characteristics of the included articles are available in Multimedia Appendix 3 [7,18-22,37,38,46-68].

In total, 12 stakeholder groups were identified in the publications. The most frequently identified stakeholder groups were nursing staff (23/31, 74%), residents (20/31, 65%), and informal caregivers (13/31, 42%). The stakeholder groups care managers (9/31, 29%), developers of surveillance technologies (7/31, 23%), physicians (5/31, 16%), LTC administrators (4/31, 13%), maintenance employees (2/31, 6%), ICT (2/31, 6%), vendors of surveillance technologies (1/31, 3%), project managers (1/31, 3%), and academics (1/31, 3%) were mentioned less frequently. A total of 9 (29%) of the 31 articles included 4 or more stakeholder groups in their research. Table 2 shows the stakeholders’ frequency of occurrence in the included articles.

**Table 2:** Frequency of stakeholder occurrence in included articles and proxy perspectives^a^.

Stakeholder	Frequency (N=31), n (%)	Proxy or partly proxy, n (%)^b^
Nursing staff	23 (74)	7 (30)
Residents	20 (65)	17 (85)
Informal caregivers	13 (42)	7 (53)
Managers	9 (29)	—^c^
Developers	7 (23)	—
Physicians	5 (16)	—
Long-term care administrators	4 (13)	—
Maintenance	2 (6)	—
ICT^d^	2 (6)	—
Vendors	1 (3)	—
Project managers	1 (3)	—
Academics	1 (3)	—

^a^Proxy perspective refers to the perspective of a stakeholder filled in by another stakeholder.

^b^Percentages in this column represent the proportion of articles in the corresponding cells in the "Frequency" column.

^c^Not applicable.

^d^ICT: information and communication technology.

### Values of Stakeholders

#### Nursing Staff

Of all identified stakeholder groups, nursing staff (registered nurses, assistant nurses, and nurse aids) were mentioned in 23 (74%) out of 31 articles. Of these 23 articles, 7 (23%) articles were written from a proxy perspective of care managers (n=4, 17%), LTC administrators (n=2, 8%), and informal caregivers (n=1, 2%).

Several values of Schwartz were represented in the stakeholder group nursing staff. The values *benevolence* and *security* were strongly represented. The value *benevolence* is related to being helpful, supporting, and assisting those in need [31]. Staff’s desire to be helpful and respond to the needs of residents also partly overlapped with the value *security,* which is characterized by striving to assure the safety of loved ones, taking precautions to prevent harm, and being warned in case of threats [45]. From the nursing staff perspective, surveillance technologies were most often applied to enhance the general safety of residents, mitigate the risk of falling, alert staff promptly, and contribute to efficient care [7,22,46]. Surveillance technologies can help nursing staff prioritize and direct their attention to where care and support are most needed [7,22,47]. Nursing staff expected monitoring technologies to increase the safety of residents [20], although they were aware of its limitations [21]. For example, they mentioned that surveillance technologies could not guarantee that residents would never fall [22]. Nursing staff rated residents’ safety higher than they rated the experience of freedom [20], possibly due to the fear of being blamed for accidents or injuries to residents [22].

Because nursing staff felt responsible for their residents, the former expressed hesitation toward entirely relying on new surveillance technologies [48]. The values *conformity* and *tradition* were reflected in the nursing staff’s conservativeness and preference to maintain their traditional routines. The nursing staff mentioned that altering their care routines was more difficult than expected. They tended to continue doing their rounds and checking on residents as usual despite the use of new technologies [49]. Managers’ proxy perspectives also recognized the difficulties in altering care routines [48]. Although nursing staff tended to keep their traditions, they were generally supportive of new technologies that contributed to improvements in daily practice, particularly when the technologies functioned as intended [46]. However, new surveillance technologies never functioned properly from the outset, and implementing them often resulted in initial malfunctions [50]. These initial malfunctions, such as false alarms, poor Wi-Fi, and slow software had a negative influence on the level of acceptance, partly due to the nursing staff’s great sense of responsibility for the residents [7,46,51]. These challenges limited the usability and accuracy of detecting unsafe situations and led to an even higher workload [7,22,46,48]. In addition, these challenges resulted in nurses’ alarm fatigue and, as a result, nursing staff primarily relied on their previous experiences with residents’ routines [21,48,51].

Moreover, nursing staff found new technologies challenging because these technologies required skills they did not previously need in their daily practice [52]. The extent to which procedures and instructions were tailored to the nursing staff’s daily work, their shifts, and professional language influenced the nursing staff’s openness to changes and the experienced extent of *self-direction* [7,20,51,53]. Moreover, they expected the technology vendors to help them, for instance, by providing support even outside regular business hours [7,51]. Nursing teams also felt supported when some of their colleagues took the lead in teaching them [7,47,51].

Although surveillance technologies contributed to providing optimal security and safety for residents, nursing staff expressed concerns about the impact of the use of monitoring technology on privacy, their competence in using the technologies, and the replacement of their roles [51,54]. This outcome relates to the value *self-direction.* Nursing staff expressed concerns that the monitoring technology was applied as a “big brother tool,” indicating a lack of confidence [7,19,37,54]. Nursing staff preferred the application of codes of ethics and limited access by authorized professionals to protect their privacy and that of residents [7,53]. In addition, staff were concerned that monitoring had an impact on resident relationships [54] and that their role would be replaced by technology [51]. The latter was also recognized by care managers, as reflected in their proxy perspectives [51,52]. Particularly, older nursing staff expressed concerns about experiencing challenges while working with technology, resulting in a reserved attitude toward care technology [51]. The extent to which nursing staff felt involved influenced the freedom they experienced to determine their ideas and actions. This outcome impacted their openness to changes, represented by the values *self-direction in action and thought*. From a proxy perspective, care managers and administrators recognized these challenges faced by nursing staff, and they mentioned that nursing staff needed time to adapt to new care technologies before they could appreciate them [55,56].

Because surveillance technologies directly interfered with the nursing process, nursing staff expressed a desire to be involved from the beginning, for instance by being involved in discussions and decision-making processes [22], to be able to express their needs, opinions, and concerns [53]. The values *achievement* and *power* are related to the nursing staff’s desire to be acknowledged and appreciated as important stakeholders.

In summary, the values *benevolence, security, conformity,* *tradition*, *self-direction in action and thought, achievement,* and *power* were represented among the stakeholder group of nursing staff.

#### Residents

Of the 31 included articles, 20 (65%) described residents as stakeholders, with 17 (85%) articles based on a (partial) proxy perspective of nursing staff (n=12, 47%), informal caregivers (n=4, 20%), LTC administrators (n=2, 10%), and developers of surveillance technologies (n=2, 10%).

Several residents who were interviewed, said they were aware of their dependency on care as a result of their cognitive decline. They adapted to life’s circumstances, reflecting the value *humility*. Moreover, they indicated that care customized to their preferences and needs supported their independence and contributed to their safety [52,55]. The value *(personal) security* is represented in the residents’ feeling of being heard by care professionals. This means that professionals know where they are, respond to their alarms, and can care for them [22,53,57]. Consequently, surveillance technologies were perceived as part of the deal, improving their safety, and receiving individualized care [38,57]. This was recognized by nursing staff because they mentioned that surveillance technologies enhanced safety and contributed to the care, in line with residents’ personal preferences, contributing to the latter’s level of independence [52,53,55].

Residents knew that they partly gave up their privacy when they moved to a nursing home. Nevertheless, surveillance technologies could contribute to their feeling of *(personal) security* because the technologies protect their privacy. Coded doors, for example, could prevent other residents from unintentionally entering their rooms [19,38]. Although residents expressed a feeling of increased *(personal) security* due to surveillance technologies, they feared the consequences of these technologies as they expressed worries that these technologies would replace the valuable human contact with staff, because social contacts were an essential and highly valued part of life [37,38].

Furthermore, the values *conformity* and *tradition* seemed important to residents. These values include the avoidance of significant changes in their living environment, for instance, due to the use of surveillance technologies. Residents emphasized the importance of maintaining a feeling of homeliness [38,57]. In addition, surveillance technology should not jeopardize their feeling of homeliness or be (too) visible. Instead, surveillance technology should be aesthetically pleasing, easy to use, and not disrupt their daily routines [57,58]. Caregivers, as proxies, noticed that the extent of devices’ intrusiveness to residents influenced the acceptability of devices [47,55,59]. Moreover, several residents expressed resistance to technology based on the usefulness they experienced [60]. The relevance of these factors was recognized in the proxy perspective of nursing staff and informal caregivers [47,59,61]. In addition, nursing staff experienced fewer nighttime disturbances for residents resulting in calmer nights [7,51]. Nursing staff noticed that the residents’ openness to changes and the experienced *self-direction* decreased when there were more false alarms [57], which was recognized in the reluctance the latter expressed and is related to the experienced usefulness.

Residents’ feelings of being stigmatized or being regarded as patients increased when their wishes regarding the visibility, appearance, and usability of surveillance technologies were not met [18]. This outcome aligns with the value *face*, which emphasizes maintaining one’s public image and avoiding humiliation. Regarding this value, residents expressed greater concern about cameras than about other devices. These concerns were particularly about being recognizable in images while performing personal and hygienic activities, evoking feelings of intrusion and vulnerability [37,38,54]. This factor was similarly mentioned by managers when they expressed their concerns about the invasion of residents’ privacy and dignity arising from surveillance cameras [54].

In summary, the values *humility*, *(personal) security, conformity, tradition, self-direction, and face* were most represented in the resident stakeholder group.

#### Informal Caregivers

Of the 31 articles, 13 (42%) mentioned informal caregivers (such as family caregivers and authorized representatives) as stakeholders. In total, 7 (53%) of these articles (partly) described a proxy perspective. Proxy perspectives were mostly represented by nursing staff (n=6, 46%), LTC administrators (n=2, 15%), and physicians (n=1, 8%).

Informal caregivers, mostly family members, expressed their concerns about the safety of their loved ones. Preventing (new) falls was often mentioned as a reason to use surveillance technology [19,22]. In their desire to contribute to the safety and well-being of their loved ones, deriving from the values *benevolence* and *security*, informal caregivers often valued residents’ personal safety above possible threats to their privacy and freedom of movement [22,38]. This was also recognized by nursing staff and physicians, who observed informal caregivers’ peace of mind when surveillance technologies were used [21,54]. In line with this value benevolence, informal caregivers felt responsible for the well-being of their loved ones. Informal caregivers feared that surveillance technologies would replace valuable human contact that arises from this value, and this fear may be reinforced by informal caregivers’ awareness of the staff shortages in nursing homes [38,51]. This fear was recognized by nursing staff who indicated that they perceived it among informal caregivers [38,51]. Informal caregivers noted surveillance technologies should support nursing staff rather than replace them [38]. From a transcendent perspective, the value *universalism* seemed to be represented in informal caregivers’ concerns about striving for equality and protection of people who were weak [31].

Informal caregivers mentioned they were willing to accept a wide range of surveillance technologies, including video surveillance, as long as they were convinced about the contribution these technologies made to the safety, quality of life, and well-being of their loved ones [22,37,38]. In addition, nursing staff and managers said that the level of informal caregivers’ openness to changes was also determined by their perception of usefulness and their interest in technologies [53]. Informal caregivers’ willingness to accept a broad range of surveillance technologies and their openness to changes reflected the value *self-direction*, embodying their ability to choose their goals and be involved in decision-making. The prerequisite of getting involved in (discussions about) applying surveillance technologies to their loved one [38,47,53,62] arises from the value *power*. Informal caregivers mentioned that these discussions should occur between all relevant stakeholders, such as residents, relatives, and nursing staff. In addition, informal caregivers wanted to be asked for formal consent as they were (authorized) representatives [38]. In practice, informal caregivers mentioned that they were not or not sufficiently informed about the available surveillance technologies [62].

In summary, the values *benevolence* and *security*, *universalism*, *self-direction, and power* were represented in the stakeholder group of informal caregivers.

#### Care Managers

Of the 31 articles, 9 (29%) mentioned care managers as stakeholders. Several care managers mentioned that their priority was to manage the 24×7 health care service and that surveillance technologies could contribute to achieving this aim [53,54]. The application of surveillance technologies increased the safety of residents, which is related to the values *benevolence* and *security*. Furthermore, data from these systems could be used to defend nursing homes against allegations of negligence leveled by families [19]. In line with this situation, care managers mentioned that data could help them monitor staff and hold them to account. However, care managers acknowledged that responding to an alarm was no guarantee that care was being provided [19]. The ethical objections against surveillance technologies that care managers mentioned were particularly aimed at the potential impact on residents’ privacy, rather than the impact on nursing staff [19,22]. They acknowledged camera surveillance could contribute to a “big brother” effect and a culture of mistrust [19,54]. For care managers, surveillance technologies particularly seemed to represent values with a personal focus, namely, to have control over and manage the residential care facility. This outcome aligned with values such as *power* and *achievement.*

Care managers mentioned they were often insufficiently prepared for new ways of working and, subsequently, the different authority structures resulting from implementation strategies. In addition, they mentioned they were unable to make implementations a priority due to other organizational priorities [51]. The unpreparedness for changes they experienced could be a consequence of the changed ways of working, fear of the unknown, and clinging to the values *conformity and tradition*. Nevertheless, they were open to changes, although they also experienced unpreparedness for cocreation from several stakeholders [51]. This unpreparedness stems from an expectation of a tailored solution from vendors—not a realization that everyone’s input including their own was a prerequisite for a joint implementation process [51]. Care managers taking the initiative in prioritizing reflections with other stakeholders to discuss dilemmas was mentioned as a facilitator for the successful use of surveillance technologies [51]. In this respect, care managers faced challenges in their *self-direction in action and thought* [31].

Furthermore, care managers felt that values *power* and *achievement* might be occasionally threatened. They indicated that they were concerned about increasing costs associated with new technologies, such as surveillance systems, while revenues remained stagnated [63]. In addition, surveillance technologies were not always as robust as they needed to be to withstand use in nursing home practice, leading to recurring costs due to damaged products [19]. Furthermore, care managers mentioned they felt restricted by rules and contractual obligations with vendors, which could hinder their access to technologies [19].

In summary, the values *benevolence* and *security* were somewhat represented in the stakeholder group of care managers. However, the values *power,* *achievement, conformity,* *tradition,* and *self-direction in action and thought* were more clearly represented.

#### Developers

A total of 7 (23%) of the 31 articles mentioned developers as stakeholders. Developers envisioned surveillance technologies would be used to enhance the safety of residents with dementia and improve the security of residents and nursing staff while respecting the privacy of both [19,55,58]. This outcome aligned with the value *security*.

In addition, developers mentioned they sought to use their technologies to enable nursing staff to support and assist residents with progressive diseases such as dementia. Due to the characteristics of the resident population, developers mentioned that surveillance technologies should be dynamic and scalable and be designed for failure and intensive use [55]. This outcome reflected the value *benevolence* as it underscored their determination to support and assist those in need*.*

Developers emphasized that by testing monitoring technologies new insights were created and improvements could be made. Testing in real life supported them to achieve success, in line with the values *achievement* and *power*. However, they acknowledged that high error rates in initial tests had an influence on the nursing staff’s openness to change [52,58,60,61]. Conversely, many initial errors occurred due to unskillful use. A higher level of training for nursing staff could reduce these errors [58]. This outcome challenged developers’ values *stimulation* and *self-direction* because they were generally excited about new technologies when they noticed the impact of their technologies on end users in nursing homes [60]. Furthermore, developers emphasized the importance of evaluating the effects of surveillance technologies in nursing homes [60]. Moreover, they underlined the importance of development in close collaboration with end users and specialists in dementia care to meet their needs and requirements [52,55]. Developers acknowledged that this close collaboration was an intensive process [52,55].

In summary, for the developer stakeholder group, the values *security, benevolence, achievement, power, stimulation,* and *self-direction* were most represented.

#### Physicians

Of the 31 articles, 5 (16%) included physicians as stakeholders in the application of surveillance technologies.

As with nursing staff and informal caregivers, the values *security* and *benevolence* were represented, as physicians agreed that providing safety was an important reason to apply these technologies. Moreover, they noted these technologies offered peace of mind to nurses and informal caregivers [21,37]. This outcome might explain physicians’ high acceptance rate of surveillance technologies [55,58].

In summary, the values *security* and *benevolence* were clearly represented in the stakeholder group of physicians.

#### LTC Administrators

LTC administrators were mentioned in 4 (13%) of the 31 articles. LTC administrators expressed their vision of being at the forefront of implementing new technologies, especially in newly constructed nursing homes. Generally, new technologies were considered an important solution for health care challenges [52]. In these administrators’ perspectives, the values *self-direction in action and thought* and *stimulation* were reflected. However, LTC administrators acknowledged that implementing these technologies challenged institution’s openness to changes because these new technologies demanded time and resources across various roles and professions [51]. LTC administrators noted that education for staff was an important issue; however, they sometimes questioned the abilities of nursing staff to master the new technologies despite offering education to the latter [37].

The values *power* and *achievement* emerged in the acknowledgment by LTC administrators that their residential care facilities were perceived as more attractive employers when they used modern surveillance technologies [37,52]. The financial investments required could force organizations to opt for a cheaper but more generic design, although this may be less suitable than preferred designs [19]. Consequently, LTC administrators could feel restricted in their control over resources. In line with care managers’ perspective, LTC administrators mentioned that organizational contracts limited their scope and flexibility around product choices and ongoing maintenance [19].

In summary, the values *self-direction in action and thought, stimulation, power,* and *achievement* were represented in the stakeholder group of LTC administrators.

#### Values of the Other Stakeholders

The other stakeholder groups, that is, maintenance staff, ICT staff, vendors of surveillance technologies, project managers, and academics were mentioned only once or twice in the 31 articles included. Maintenance staff mentioned that although new surveillance technologies might seem to have a limited scope, only affecting night shift workers and residents, these technologies could also have an influence on janitors, cleaning staff, and substitute personnel [37,51]. In their view, maintenance staff members were also in need of information and education to accommodate the technologies in their (cleaning and maintenance) routines. When they were not informed or did not receive education, they reported difficulties, for example, in replacing sensors and reconnecting cables after cleaning [37,51]. Because a new system is only as good as the people who are responsible for operating it, the people operating the system could affect its reliability [37,51]. Maintenance staff expressed a desire to be acknowledged as important stakeholders and be involved in education, which relates to their openness to changes in the values *self-direction* and *stimulation*. In line with the apprehensions of nursing staff, maintenance staff expressed concerns about being observed [37]. For instance, maintenance staff sometimes felt threatened due to their openness to changes and *self-direction*, reducing their motivation to engage with new technologies.

ICT staff emphasized the importance of system and component interoperability for ensuring system reliability because new systems were often installed into existing systems and infrastructure [19,51]. However, they mentioned that this interoperability between systems often was not facilitated by manufacturers [19]. Furthermore, the staff highlighted the importance of their involvement from the outset in exploring this compatibility and interoperability to prevent compromises in residents’ safety and security [51]. This outcome reflected the need for ICT staff to be involved and acknowledged as stakeholders, in line with the values *power* and *achievement*. However, in practice, it was seen that ICT staff only became seriously involved when systems were unstable or errors occurred [51].

In the research by Dugstad et al [51], vendors of surveillance technologies and ICT were challenged to adopt a more socially focused approach to bridge differences between stakeholder groups. The vendors mentioned that despite their knowledge about their products, they needed the nursing staff’s insights to ensure their technologies worked in specific care environments due to the great variety of care practices and infrastructure. Besides vendors striving for their own success, reflecting the values *power* and *achievement*, they were challenged to pursue a higher purpose, in line with the value *universalism. *Universalism relates to vendors’ willingness to engage with other stakeholders to contribute to successful applications. In line with this value, ICT staff and vendors recognized being challenged to adopt a language that was more understandable for nursing staff who experienced differences in professional cultures and language (jargon) [51]. The ICT staff and vendors mentioned they were not used to adapting their language; therefore, misunderstandings were initially manifested due to a lack of knowledge and insight into each other’s workflows. This situation challenged them to transcend their profession and jargon in order to contribute to a successful digital transformation [51]. Local project managers also underlined the importance of recognizing and bridging cultural differences as conditions for ensuring successful digital transformation. Hence, project managers identified their role as translators between different stakeholders [51], in line with the value *universalism*.

Finally, academics mentioned that the balance between freedom and security is important. They tended to value freedom of movement over security [20]. The socially focused value *benevolence* was reflected in being devoted to the welfare of others and protecting people with an increased care dependency. However, academics tend to attach great importance to residents’ freedom to determine their own actions despite the dementia, which relates to the *value self-direction*.

**Figure 2:**
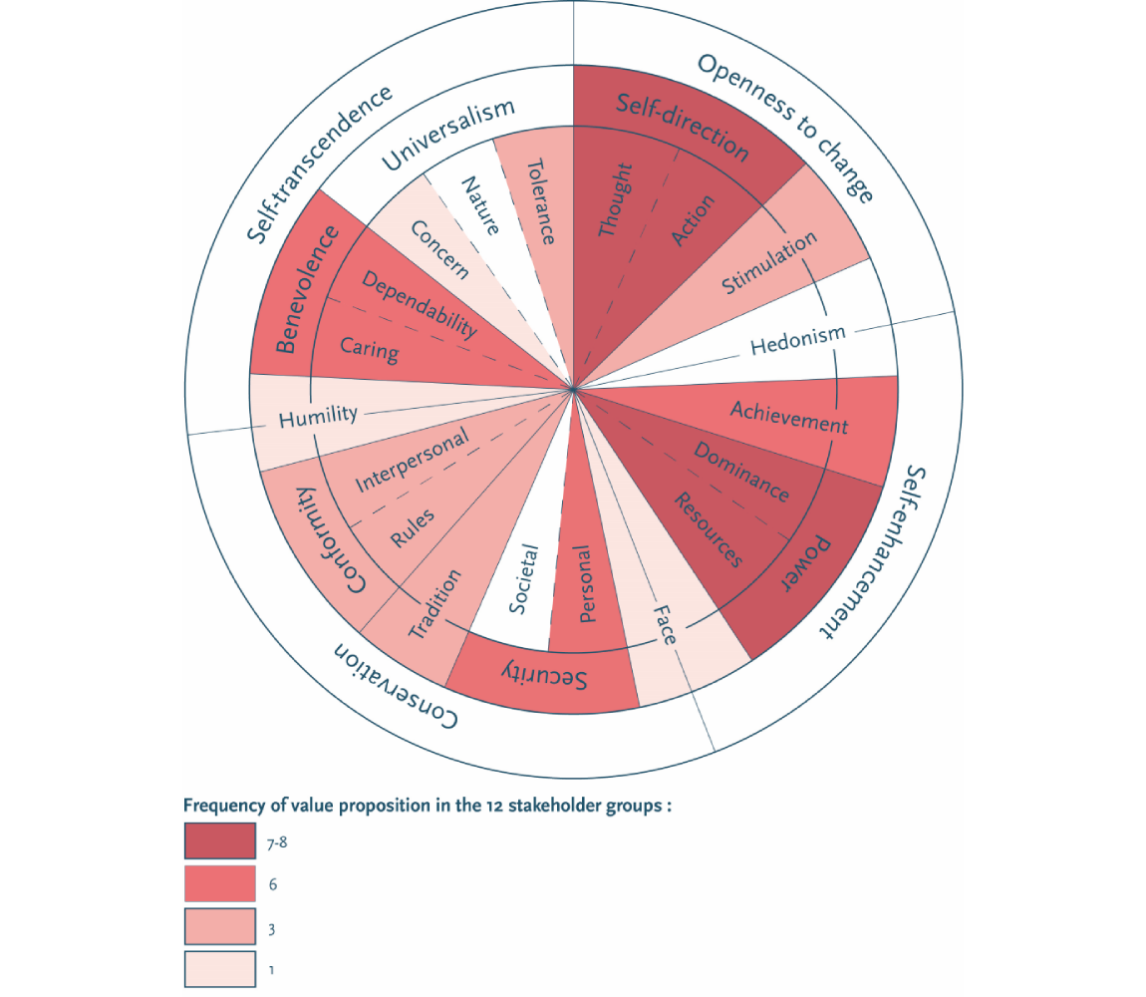
Value palette for all stakeholders in the proposed circular structure of the 19 basic human values by Schwartz [32].

Figure 2 presents the stakeholders’ basic human values. The darker the color scheme, the more often this value is applied to various stakeholders.

## Discussion

### Principal Findings

This scoping review aimed to identify the stakeholders involved in applying surveillance technologies for people with dementia in nursing homes and to describe the values of these stakeholders. Overall, 12 stakeholder groups were identified in 31 articles. The stakeholder groups of nursing staff, residents, and informal caregivers were most often mentioned in these articles. Several stakeholder groups, such as physicians, LTC administrators, maintenance staff, ICT staff, vendors, and project managers, were mentioned significantly less frequently [51,52,55].

Although many stakeholder groups emphasized the importance of being acknowledged and feeling involved as stakeholders [22,37,51,53,62], they said that their involvement should be improved [22,51,53,62]. Moreover, several stakeholders mentioned feeling dissatisfied when they did not feel sufficiently involved [53,62]. As dissatisfaction could detract from success, all relevant stakeholders should be involved [53]. Furthermore, in literature, the importance of involving both primary stakeholders, also known as end users, and secondary stakeholders (ie, more distantly involved stakeholders) is underlined as contributing to the successful implementation of care technologies, such as surveillance technologies [22,28,29].

To determine which primary and secondary stakeholders should be involved and engaged, it is important to identify these stakeholders [69]. Identifying stakeholders can be accomplished, for example, through a stakeholder analysis [69]. Several stakeholder matrices such as the power-interest matrix, the 3D matrix, and the responsible, accountable, consulted, and informed matrix offer tools to identify stakeholders and categorize their attributes such as their power, position, and level of interest [69,70]. In addition, these matrices support prioritizing who should be involved and to what extent. These matrices reflect the increased recognition of how the characteristics of stakeholders influence innovation and implementation processes [69,71,72]. Regardless of the distinction between stakeholders’ level of involvement and their responsibilities, the resistance of any stakeholder can have an influence on the success of the implementation of care technologies.

Resistance by stakeholders is a challenge, necessitating the identification of alternative perspectives to a situation [73-75]. Therefore, besides identifying stakeholders when applying care technology, it is necessary to realize that care technologies strongly influence the care process and everyone involved. Therefore, cooperation between stakeholders who have previously rarely cooperated is essential. Hence, it is important to thoroughly know and acknowledge the different stakeholders and their differences and respect their differing interests [29].

Several implementation theories provide theoretical support to implementation processes, especially in complex contexts such as health care. One of the theoretical constructs for involving multiple stakeholders in implementation processes in health care is the normalization process theory (NPT) by May et al [76]. NPT provides tools to enhance an understanding of the social processes of thinking, enacting, and organizing work to implement and adopt interventions in care processes within health care organizations [77]. NPT distinguishes 4 constructs: coherence, cognitive participation, collective action, and reflexive monitoring. These 4 constructs emphasize the relevance of knowledge about the value that stakeholders assign to care technology and attention to stakeholders’ willingness to participate and cooperate. NPT also focuses on keeping people engaged during the whole implementation process, including reflecting upon and appraising the effect of newly implemented technology [29,78].

A common thread across the NPT as well as other implementation theories is the need for cooperation and communication between stakeholders focusing on knowing and acknowledging each other’s perspectives, values, and interests [29,79]. For instance, in the early 90s, Gregory and Keeney [72] wrote about the right of multiple stakeholders to be involved in policy decision processes and consequently balance between conflicting objectives. In addition, they mentioned that values, beliefs, cooperative potential, and stakeholders’ concerns are known to influence the outcome of innovation processes [72]. Similarly, in business ethics, Dentoni et al [80] mentioned the complexity of multiple stakeholder involvement in dynamic settings because each stakeholder group has its own set of values, perceptions, and interests that may clash. This situation requires fundamentally different approaches that demand understanding differing values, complex settings, and acting upon uncertain knowledge [80].

The active participation of stakeholders challenges them to effectively collaborate with a critical and open stance toward their perspectives and values. Several stakeholders in this scoping review mentioned that this collaboration demands not only time and effort but also challenges them to adapt their jargon to interprofessional cultures and bridge their differences [51,52]. Although all stakeholders should be able to operate and communicate across boundaries between different practices with each other, collaborating with people is difficult and can lead to tensions and misunderstandings related to values and interests [81]. Such collaboration demands competence to perceive differences as learning opportunities and to cross boundaries between multiple stakeholders [81,82]. Collaboration between stakeholders is receiving increasing attention from organizations. In addition, organizations have shown a growing interest in creating value through participation and interaction with multiple stakeholders. However, until now, most of the attention has been given to creating value *for* stakeholders and not *with* them. In cases of increased awareness about stakeholders’ input, values and interests may be well identified [83]. Thus, besides merely identifying and superficially involving stakeholders, it is important to pay attention to their perspectives, values, and interests.

Therefore, this scoping review also focused on what is already known about the stakeholder group values identified in the 31 articles reviewed. Several values in the theory of basic human values by Schwartz et al [31] were frequently represented among the stakeholder groups. The values *benevolence* and *security* were represented in 6 (50%) out of 12 stakeholder groups. This outcome is unsurprising given the progressive nature of dementia. Most stakeholder groups mentioned they experienced a feeling of being responsible for caring for residents with dementia and responding to their needs, in line with the value *benevolence* [21,22,37,38,55]. Concerning the value *security*, striving for safety for residents with dementia is often mentioned as a reason to apply surveillance technologies [7,22,46]. However, the meaning assigned to this value varies among the different stakeholder groups. The trade-off between safety and aspects such as privacy and freedom of movement differs among various stakeholders and appears to be related to how closely a stakeholder is involved with a resident [20]. For nursing staff, *security* is related to enhancing the safety of residents and mitigating their risk of falling [7,22,46]. This relates to the responsibility they feel for their residents because they feel accountable for accidents or injuries of residents [22,48]. For care managers, the value *security* is also related to their accountability for providing care, managing the residential care facility, assuring families that care is provided, and monitoring staff [19,54]. For residents, *security* is related to the feeling of being heard regarding their personal preferences and (care) needs and experiencing a feeling of homeliness [22,52,53,57]. Informal caregivers have concerns about their loved ones and they seem to experience more peace of mind when surveillance technologies are applied [21,54]. Consequently, informal caregivers often value safety above freedom of movement and the possible threats to privacy [19,22]. In contrast, academics, a group of stakeholders who do not have a close relationship with residents, tend to value freedom of movement above safety, giving high importance to the residents’ experience of freedom and self-determination [20].

As is evident in the considerations regarding safety, surveillance technologies that are applied in practice affect the work and living environment of several stakeholder groups [37,51,53]. Accordingly, many stakeholders emphasized their desire to be and to feel involved in the application of surveillance technologies [22,53]. Arising from this desire, the values *self-direction in action and thought* were represented in 8 (67%) of 12 stakeholder groups. This outcome underlines the relevance of thoroughly involving stakeholders throughout the implementation and application process. In addition, the degree of stakeholders’ openness to changes, their experienced *self-direction*, and their tendency to cling to traditional routines seem to be related to the extent to which they feel involved. The more they feel involved, the more they are open to changes and willing to collaborate [22,38,47,53,62]. Being and feeling involved is especially an important issue for nursing staff because surveillance technologies directly interfere with the nursing process. Therefore, nursing staff were the most frequently cited stakeholders. They were mentioned in 23 (74%) of the 31 articles. Accordingly, they expected to be involved from the beginning; asked to express their needs, opinions, and concerns [53]; involved in decision-making; and acknowledged as important stakeholders [22]. Informal caregivers mentioned that they were willing to accept a broad range of technologies for their relatives as long as the former were informed about the surveillance technologies, were involved in deliberations about the technologies, and were asked for their consent regarding the application of the technologies [38,47,53,62].

Related to the value *self-direction* were the values *power* and *achievement*, representing stakeholders’ desire to exert their influence on other people or use material resources and pursue success in competencies and performance. The values *power* and *achievement* were mentioned by 7 and 6 stakeholder groups, respectively. These values are a reflection that stakeholders such as nursing staff, informal caregivers, developers, ICT staff, and vendors want to feel recognized in their knowledge and experiences, and want to exert their influence in the application of surveillance technologies [22,38,51-53,61,62]. For care managers and LTC administrators, the values *power* and *achievement* are related to control over and the management of residential care facilities [53,54].

Although several values such as benevolence, security, self-direction, power, and achievement were represented by most of the stakeholders, the various stakeholder groups assigned different meanings to these values. Knowing and understanding diverse stakeholders’ perspectives and attitudes, including the different meanings they attribute to values, is crucial because this knowledge and understanding influence the adoption and use of technologies [84]. Integrating multiple perspectives is valuable to fully understand the complexities of care practices [29] and dementia care technology [84]. In addition, it is challenging to distinguish whether stakeholders give meaning to a certain value based on their interests or whether they act from the resident’s perspectives. This situation demands that designated persons in health care organizations have courage and take the lead in initiating meaningful and in-depth conversations where the diverse stakeholders will be challenged to communicate across their boundaries, looking beyond their own perspectives.

### Methodological Considerations

A proxy perspective was often observed in the stakeholder groups of nursing staff, residents, and informal caregivers. This outcome is not unusual because proxy perspectives often originate from stakeholders with whom one works or lives [85]. In addition, proxy perspectives are often seen in stakeholder groups that collaborate and deal with matters that touch on values and interests. Hence, a proxy perspective was regularly seen among nursing staff and informal caregivers in this scoping review. Furthermore, when making assumptions, people are less understanding of others’ actual motivations [85]. Accordingly, Kloos et al [86] researched the well-being of residents and found that nursing staff tended to overestimate the well-being of residents. Their study underlines the importance of combining proxy assessments with self-reports whenever possible. In addition, Kunicki et al [87] noted that the level of involvement of proxies and their sense of well-being could influence their perception of the resident’s preferences. Kunicki et al [87] recommended that proxies find methods to better understand residents’ preferences when residents were not able to express their preferences properly [87]. This scoping review raises the question of whether proxy perspectives of residents with dementia reveal resident’s perspectives. Although it is challenging to interview residents with dementia due to the impairments related to their condition, it is possible to involve them. Therefore, in future research, residents should be made participants in research to understand their perspectives [88,89].

### Strengths and Limitations

Previous studies primarily focused on facilitating and limiting factors and ethical dilemmas when applying surveillance technologies to people with dementia [18,23,36-38]. However, this scoping review is the first to explore which universally recognized human values are reflected in the experiences and opinions of stakeholders. Because values reflect human thinking and determine attitude and behavior, it is crucial to consider the underlying values that explain stakeholders’ behavior and reactions [34,35,79]. Stakeholders’ values were classified using the basic human values model by Schwartz. Although this model offers an empirically tested theory to predict attitudes and behaviors in different contexts and situations, it is possible that not all opinions and experiences of stakeholders could be categorized using the model. Furthermore, it is likely that the stakeholders’ values do not provide a complete picture of the values that stakeholders hold in practice because the representation of stakeholders’ values in this scoping review is based on information that did not primarily focus on stakeholders’ values.

### Recommendations for Research

Although this scoping review identified 12 stakeholder groups, most (26/31, 84%) of the articles included in the review described only 4 or fewer stakeholder groups. Therefore, various stakeholder groups were underrepresented. Future research should emphasize the involvement of all relevant stakeholders. In addition, our explorations of stakeholders’ values revealed there is insufficient information about stakeholders’ values. Hence, more research about the values that influence stakeholders’ actions and decisions should be conducted. In exploring stakeholders’ perspectives and values, a proxy perspective should be avoided where possible. In future research, inviting stakeholders to look beyond their perspectives and boundaries could be useful in mitigating language and knowledge boundaries between different stakeholders. This approach could facilitate constructive cooperation between stakeholders. In addition, efforts should be made to include residents with dementia in research to explore their perspective rather than assumptions about residents’ perspectives being largely based on a proxy perspective. Listening to people with dementia can enhance the quality, relevance, and impact of dementia research, which contributes to the enhancement of knowledge based on what we learn *from* them and their informal caregivers, in order to create knowledge *with* them [90]. Participatory research could apply to this kind of research about complex dynamic subjects; research should be conducted with rather than about stakeholders.

### Conclusions

All stakeholders involved in applying surveillance technologies expressed a desire for their perspectives and values to be acknowledged. This desire stems from the human need to be acknowledged and appreciated. Moreover, all the stakeholders manifested a willingness to be engaged and participate. The broad acknowledgment and involvement of stakeholders and an understanding of their perspectives and values contribute to the successful implementation and application of surveillance technologies for people with dementia in nursing homes. Therefore, when applying surveillance technologies for people with dementia, residential care facilities are expected to intensively collaborate with an increasing number of stakeholders. Therefore, stakeholders’ active engagement, with attention to everyone’s perspectives and values, is more important than ever.

## Appendix

Multimedia Appendix 1 PRISMA-ScR checklist.

Multimedia Appendix 2 Overview of the search string for the 5 databases.

Multimedia Appendix 3 Mapping overview of the included articles.

## Abbreviations

ICT: information and communication technology
LTC: long-term care
NPT: normalization process theory
PRISMA-ScR: Preferred Reporting Items for Systematic Reviews and Meta-Analyses Extension for Scoping Reviews
