# Supplementary material for: Values of Stakeholders Involved in Applying Surveillance Technology for People With Dementia in Nursing Homes: Scoping Review
Source: JMIR Aging. 2025 Mar 20;8:e64074. doi: 10.2196/64074 (PMC11969125; doi:10.2196/64074)
Supplement: Multimedia Appendix 2 [file aging_v8i1e64074_app2.docx]

Supplementary material

1 Search string

Search string Daniëlle van Gaans, PhD student, developed with help of Christa Niehot, information Specialist for scoping review: Surveillance technology in psychogeriatric wards: a scoping review on the values of involved stakeholders

**Medline (EBSCOhost)**

((MH "Nursing Homes+" OR MH "Long-Term Care" OR MH "Geriatrics" OR MH "Homes for the Aged") OR (TI(nursing-home* OR ((long-term OR longterm OR residental*) N3 (care OR caring OR facilit*)) OR gerontol* OR psychogeriat* OR geriatr* OR ((home OR resident*) N3 (aged)) OR ((care) N3 (home* OR elder*)))) OR (AB(nursing-home* OR ((long-term OR longterm OR residental*) N3 (care OR caring OR facilit*)) OR gerontol* OR psychogeriat* OR geriatr* OR ((home OR resident*) N3 (aged)) OR ((care) N3 (home* OR elder*))))) **AND** ((MM "Remote Sensing Technology" OR MM "Geographic Information Systems") OR (TI(camera* OR videocamera* OR global-position* OR ((locat*) N3 (track* OR technol*)) OR ((motion*) N3 (sensor*)) OR ambulatory-monitor* OR home-automation OR ((tag*) N3 (implant* OR electronic* OR care)) OR grannycam* OR granny-cam* OR ((healthcare* OR assist* OR care) N3 (technol*)) OR domotic* OR artificial-intelligen* OR AI OR gerontechnolog* OR geron-technolog* OR ((surveillan* OR infrared*) N3 (technol* OR video* OR electronic*)) OR ((video*) N3 (monitor*)) OR big-brother* OR ((tag* OR track* OR locat* OR data) N12 (GPS)) OR ((track*) N3 (technol* OR tag*)) OR ((tag* OR track*) N3 (system*)) OR ((monitoring) N3 (technol* OR bed OR bed OR system*)) OR ((welfare*) N2 (technol*)))) OR (AB(camera* OR videocamera* OR global-position* OR ((locat*) N3 (track* OR technol*)) OR ((motion*) N3 (sensor*)) OR ambulatory-monitor* OR home-automation OR ((tag*) N3 (implant* OR electronic* OR care)) OR grannycam* OR granny-cam* OR ((healthcare* OR assisti* OR care) N3 (technol*)) OR domotic* OR artificial-intelligen* OR AI OR gerontechnolog* OR geron-technolog* OR ((surveillan* OR infrared*) N3 (technol* OR video* OR electronic*)) OR ((video*) N3 (monitor*)) OR big-brother* OR ((tag* OR track* OR locat* OR data) N12 (GPS)) OR ((track*) N3 (technol* OR tag*)) OR ((tag* OR track*) N3 (system*)) OR ((monitoring) N3 (technol* OR bed OR bed OR system*)) OR ((welfare*) N2 (technol*)))))

2252 results

**NOT** ((MM "Telemedicine" OR (TI(telemedicine OR community-dwel* OR living-at-home* OR telehealth* OR blockchain* OR humanized-robot* OR robot OR robots OR robotic-car* OR home-care* OR homecare*)) OR (AB(telemedicine OR community-dwel* OR living-at-home* OR telehealth* OR blockchain* OR humanized-robot*)))

1676 results

**NOT** (PT "Editorial" OR PT "Comment" OR PT "Letter" OR PT "News" OR PT "Congress" OR PT "Meeting Abstract" OR PT "Abstracts" OR PT "Academic Dissertation" OR PT "Published Erratum" OR PT(book* OR chapter* OR dissertation abstract*)) NOT ((MH "Systematic Reviews as Topic" OR PT "Systematic Review") OR TI(systematic-review* OR scoping-review*))

**CINAHL**

((MH "Nursing Homes+" OR MH "Long Term Care" OR MH "Geriatrics" OR MH "Residential facilities" OR “nursing home patients”) OR (TI(nursing-home* OR ((long-term OR longterm OR residental*) N3 (care OR caring OR facilit*)) OR gerontol* OR psychogeriat* OR geriatr* OR ((home OR resident*) N3 (aged)) OR ((care) N3 (home* OR elder*)))) OR (AB(nursing-home* OR ((long-term OR longterm OR residental*) N3 (care OR caring OR facilit*)) OR gerontol* OR psychogeriat* OR geriatr* OR ((home OR resident*) N3 (aged)) OR ((care) N3 (home* OR elder*))))) **AND** ((MM "Assistive technology" OR “assistive technology services” OR MM "Geographic Information Systems") OR (TI(camera* OR videocamera* OR global-position* OR ((locat*) N3 (track* OR technol*)) OR ((motion*) N3 (sensor*)) OR ambulatory-monitor* OR home-automation OR ((tag*) N3 (implant* OR electronic* OR care)) OR grannycam* OR granny-cam* OR ((healthcare* OR assist* OR care) N3 (technol*)) OR domotic* OR artificial-intelligen* OR AI OR gerontechnolog* OR geron-technolog* OR ((surveillan* OR infrared*) N3 (technol* OR video* OR electronic*)) OR ((video*) N3 (monitor*)) OR big-brother* OR ((tag* OR track* OR locat* OR data) N12 (GPS)) OR ((track*) N3 (technol* OR tag*)) OR ((tag* OR track*) N3 (system*)) OR ((monitoring) N3 (technol* OR bed OR bed OR system*)) OR ((welfare*) N2 (technol*)))) OR (AB(camera* OR videocamera* OR global-position* OR ((locat*) N3 (track* OR technol*)) OR ((motion*) N3 (sensor*)) OR ambulatory-monitor* OR home-automation OR ((tag*) N3 (implant* OR electronic* OR care)) OR grannycam* OR granny-cam* OR ((healthcare* OR assisti* OR care) N3 (technol*)) OR domotic* OR artificial-intelligen* OR AI OR gerontechnolog* OR geron-technolog* OR ((surveillan* OR infrared*) N3 (technol* OR video* OR electronic*)) OR ((video*) N3 (monitor*)) OR big-brother* OR ((tag* OR track* OR locat* OR data) N12 (GPS)) OR ((track*) N3 (technol* OR tag*)) OR ((tag* OR track*) N3 (system*)) OR ((monitoring) N3 (technol* OR bed OR bed OR system*)) OR ((welfare*) N2 (technol*)))))

1706 results

**NOT** ((MM "Telemedicine" OR (TI(telemedicine OR community-dwel* OR living-at-home* OR telehealth* OR blockchain* OR humanized-robot* OR robot OR robots OR robotic-car* OR home-care* OR homecare*)) OR (AB(telemedicine OR community-dwel* OR living-at-home* OR telehealth* OR blockchain* OR humanized-robot*)))

1264 results

**NOT** (PT "Edit and review" OR PT "Abstracts" OR PT "Theses and dissertations" OR PT(book* OR chapter* OR dissertation abstract*)) NOT ((MH "Systematic Reviews" OR PT "Systematic Review") OR TI(systematic-review* OR scoping-review*))

**PsycINFO**

((SU "Nursing Homes+" OR SU "Long-Term Care" OR SU "Geriatrics" OR SU "nursing home residents") OR SU "Residential Care Institutions" OR (TI(nursing-home* OR ((long-term OR longterm OR residental*) N3 (care OR caring OR facilit*)) OR gerontol* OR psychogeriat* OR geriatr* OR ((home OR resident*) N3 (aged)) OR ((care) N3 (home* OR elder*)))) OR (AB(nursing-home* OR ((long-term OR longterm OR residental*) N3 (care OR caring OR facilit*)) OR gerontol* OR psychogeriat* OR geriatr* OR ((home OR resident*) N3 (aged)) OR ((care) N3 (home* OR elder*))))) **AND** ((SU "Navigation technology" OR GPS) OR (TI(camera* OR videocamera* OR global-position* OR ((locat*) N3 (track* OR technol*)) OR ((motion*) N3 (sensor*)) OR ambulatory-monitor* OR home-automation OR ((tag*) N3 (implant* OR electronic* OR care)) OR (grannycam* OR granny-cam*) OR ((healthcare* OR assist* OR care) N3 (technol*)) OR domotic* OR (artificial-intelligen* OR AI) OR gerontechnolog* OR geron-technolog* OR ((surveillan* OR infrared*) N3 (technol* OR video* OR electronic*)) OR ((video*) N3 (monitor*)) OR big-brother* OR ((tag* OR track* OR locat* OR data) N12 (GPS)) OR ((track*) N3 (technol* OR tag*)) OR ((tag* OR track*) N3 (system*)) OR ((monitoring) N3 (technol* OR bed OR bed OR system*)) OR ((welfare*) N2 (technol*)))) OR (AB(camera* OR videocamera* OR global-position* OR ((locat*) N3 (track* OR technol*)) OR ((motion*) N3 (sensor*)) OR ambulatory-monitor* OR home-automation OR ((tag*) N3 (implant* OR electronic* OR care)) OR (grannycam* OR granny-cam*) OR ((healthcare* OR assisti* OR care) N3 (technol*)) OR domotic* OR (artificial-intelligen* OR AI) OR gerontechnolog* OR geron-technolog* OR ((surveillan* OR infrared*) N3 (technol* OR video* OR electronic*)) OR ((video*) N3 (monitor*)) OR big-brother* OR ((tag* OR track* OR locat* OR data) N12 (GPS)) OR ((track*) N3 (technol* OR tag*)) OR ((tag* OR track*) N3 (system*)) OR ((monitoring) N3 (technol* OR bed OR bed OR system*)) OR ((welfare*) N2 (technol*)))))

1007 results

**NOT** ((SU "Telemedicine" OR (TI(telemedicine OR community-dwel* OR living-at-home* OR telehealth* OR blockchain* OR humanized-robot* OR robot OR robots OR robotic-car* OR home-care* OR homecare*)) OR (AB(telemedicine OR community-dwel* OR living-at-home* OR telehealth* OR blockchain* OR humanized-robot*)))

791 results

**NOT** ((PT(book* OR chapter* OR dissertation abstract*)) NOT ((SU "Systematic Reviews" OR PT "Systematic Review") OR TI(systematic-review* OR scoping-review*))

**ACM**

Two separate searches: 1 field title, 1 field abstract

Abstract:

"nursing home" OR "nursing homes" OR "long term care" OR "longterm care" OR "long term caring" OR "longterm caring" OR "long term facility" OR "longterm facility" OR "long term facilities" OR "longterm facilities" OR gerontol* OR psychogeriat* OR geriatr* OR aged OR "care home" OR "care homes" OR "elder care"

6871 results

+

Abstract:

camera* OR videocamera* OR "global position" OR "location tracking" OR "location technology" OR "location technologies" OR "motion sensor" OR "motion sensors" OR "ambulatory monitoring" OR "home automation" OR tagging OR grannycam* OR "granny cam" OR "granny cams" OR "healthcare technology" OR domotic* OR "artificial intelligence" OR AI OR gerontechnolog* OR "geron technology" OR "surveillance technology" OR "surveillance technologies" OR "surveillance video" OR "electronic surveillance" OR "surveillance videos" OR "electronic surveillances" OR “surveillanc*” OR "infrared technology" OR "infrared video" OR "infrared videos" OR "infrared technologies" OR "video monitoring" OR "big brother" OR GPS OR "tracking technology" OR "tracking technologies" OR "tracking system" OR "tracking systems" OR tagging OR "welfare technology" OR "welfare technologies" OR “sensor*”OR “assist*”

**IEEE**

After search choose for Journals and Early access articles

"nursing home" OR "nursing homes" OR "long term care" OR "longterm care" OR "long term caring" OR "longterm caring" OR "long term facility" OR "longterm facility" OR "long term facilities" OR "longterm facilities" OR gerontol* OR psychogeriat* OR geriatr* OR aged OR "care home" OR "care homes" OR "elder care"

**AND**

camera* OR videocamera* OR "global position" OR "location tracking" OR "location technology" OR "location technologies" OR "motion sensor" OR "motion sensors" OR "ambulatory monitoring" OR "home automation" OR tagging OR grannycam* OR "granny cam" OR "granny cams" OR "healthcare technology" OR domotic* OR "artificial intelligence" OR AI OR gerontechnolog* OR "geron technology" OR "surveillance technology" OR "surveillance technologies" OR "surveillance video" OR "electronic surveillance" OR "surveillance videos" OR "electronic surveillances" OR “surveillance*” OR "infrared technology" OR "infrared video" OR "infrared videos" OR "infrared technologies" OR "video monitoring" OR "big brother" OR GPS OR "tracking technology" OR "tracking technologies" OR "tracking system" OR "tracking systems" OR tagging OR "welfare technology" OR "welfare technologies" OR “sensor” OR “assist”
