# Supplementary material for: Values of Stakeholders Involved in Applying Surveillance Technology for People With Dementia in Nursing Homes: Scoping Review
Source: JMIR Aging. 2025 Mar 20;8:e64074. doi: 10.2196/64074 (PMC11969125; doi:10.2196/64074)
Supplement: Multimedia Appendix 3 [file aging_v8i1e64074_app3.docx]

Supplementary material

2. Mapping overview of the included articles (n=31)

| **Author/ Title** | **Setting** | **Participants / duration study** | **Type Surveillance technology** | **Aim** | **Stakeholders mentioned** | **Outcome** | **Study Design** |
| --- | --- | --- | --- | --- | --- | --- | --- |
| Abbate (2014)  Canada  *Usability study of a wireless monitoring system among Alzheimer's disease elderly population* | Nursing Home (n=1) in News Brunswick | 4 people affected by Alzheimer dementia, MMSE <12  1 month field test | A minimally invasive monitoring sensor: aim to detect falls and preconditions.  Fall detector worn near waist, headband to capture brain activity, ambient sensors in bed, chair, door, toilet and a camera based monitoring system | Testing the usability and acceptability of wearable monitoring system in people with dementia | Residents (proxy from developers)  Developers= Technicians | Ergonomic and aesthetic modifications are necessary to improve the level of usability and acceptability of wearable monitoring sensors, especially in an elder population | Exploratory study/ qualitative research design |
| Al-Oraibi (2012)  Norfolk, UK  *Impact and economic assessment of assistive technologies in care homes in Norfolk, UK* | Care home 1 (CH 1): Rehabilitation and residential focussed care home  Care home 2 (CH 2): Care home for people with advanced dementia | 70 participants (residents) of residential and rehabilitation focussed home  CH 1: 10 months before and 10 months after start ST  CH 2: 6 months before and 6 months after start ST | Assistive technology (AT) system consists of: pull cord/ pendent alarm/ passive infrared movement sensor, flood detector, urethra sensor, pressure pad/ mat bed and chair, speech unit, control/ response software on computer | To examine whether new installed AT system in care homes reduced the fall-incidents and the demand for health services due to a fall | Data collection from incident reports/ stakeholder management | In people with advanced dementia overall there was an increase of fall incidents after applying new AT system, costs per incident increased, in people from care home 1 (rehabilitation and dementia) costs per incident decreased  Differences in fall incidents and costs appear to be related to level of dementia | Quantitative study, retrospective case control |
| Aloulou (2013)  Singapore  *Deployment of assistive living technology in a nursing home environment methods and lessons learned* | Nursing home (n= 1) in Singapore | Trial with 8 clients with moderate dementia and 2 caregivers  Period data collection: pre-deployment 3 months  Trial 14 months | Ambient assistive living solution: low-cost and non-intrusive sensors (sensor: pressure, proximity, vibration, motion), devices for interaction: speakers, tags in bracelets to locate, fall detection, tablets for residents, smart phones for nurses | To present the approach they have adopted, to develop and deploy a system for ambient assistive living in a nursing home/ evaluate its performance and usability in real world setting as opposed to prototype testing in laboratories | Resident perspective  Nursing staff perspective  Technician perspective/ developers  Managers  Physicians | Keywords:  Development, testing and evaluating AAL systems with diverse stakeholders (multidisciplinary design) in real life setting is helpful/ extracts lessons learned  Respecting privacy is major requirement  Professional caregivers need this kind of assistance mostly in bedrooms (most time spent) and during the night when there are less caregivers | Quantitative design/ trial in real life setting/ |
| Anker-Hansen (2022)  Norway  *Informal caregivers and assistive technology in Norwegian nursing homes* | Nursing homes (n=2) in Norway, all included wards were for people with dementia | 11 participants, informal caregivers for a resident in one of the two included nursing homes | Different types of assistive technologies such as code locked doors, tablets, activity bike, monitoring technologies such as GPS and other localization technology, sensor for lights, wrist watch with alarm. | To explore informal caregivers experiences and perspectives concerning assistive technologies (AT) in two nursing homes through the conceptual lens of person-centeredness | Informal caregivers | Informal caregivers had a lack of knowledge and information regarding the existing AT, they wanted more information. They expressed a desire for AT to increase activity and safety which promotes dignity, quality of life and quality of care for their family member | Qualitative research design |
| Bankole (2011)  USA, Virginia  *Continuous non invasive assessment of agitation in dementia using inertial body sensors* | Nursing home (n= 1), a dementia unit | 6 participants with dementia (MMSE 0-11) identified as being at risk for agitated behaviour  Period data collection: 6 weeks | Wireless body sensor network (BSN), TEMPO 3, Of eight body sensors three accelerometers were used: on dominant wrist, waist and opposite leg.  *Gyroscopes were turned off* | To explore the ability of a custom inertial wireless body sensor network (BSN) to objectively detect and quantify agitation, by objectively, continuously and non-invasively measurement of movement in older dementia people validating against clinical measures at people with dementia within the nursing home sector | Residents: proxy from observations of researchers and proxy by family members and caregivers  Family caregivers: Proxy conclusion from developers  Nursing staff: Proxy conclusion from developers  Developers | Clients: seemed to tolerate devices, they could remove the Tempo nodes, they tend to ‘tiddle’ with things within reach  Family: about resistance seen in residents  Prof. Caregiver: about resistance seen in residents  Developers: BSN’s (body sensor nodes) have the potential for agitation assessment, at least aggressive agitation | Quantitative  Repeated measures design |
| Berridge (2019)  USA  *Cameras on beds: the ethics of surveillance in nursing homes* | Members of the American Health Care Association (AHCA) and National Center for Assisted Living (NCAL) in 2016 | Survey, 273 participants from 39 states | Camera surveillance | To gain insight in the ethical implications of how camera-surveillance is used in nursing homes and assisted living facilities to keep older residents safe | Residents: proxy from managers/ administrators  Informal caregivers: proxy from managers/ administrators  Nursing staff: proxy from managers/ administrators  Management | Majority (75%= 172) of respondents thinks privacy of residents is invaded, also concern to roommates, staff and visitors privacy,  With camera use concerns about:  Dignity of residents  Can impede care relationships/  Person centered care  Privacy invasion  No home-like experience  Potential to demoralize, offend, stress, intimidate, show lack of confidence, culture of mistrust | Quantitative research design |
| Bourbonnais (2019)  Canada  *Conditions and ethical challenges that could influence the implementation of technologies in nursing homes* | Nursing homes (n=5) in Montreal, most people have severe cognitive impairment | Semi structured interviews with:  9 participants 9 care- managers, 3 family caregivers, 8 formal caregivers | An intelligent video monitoring system (IVS) that consists of small closed-circuit cameras, The IVS monitors can automatically detect a fall or responsive behaviour | To explore the conditions that may influence the implementation of an interactive mobile application (app) and an intelligent video monitoring system (IVS) in nursing homes (NHs) and the ethical challenges of their use | Resident: proxy from informal caregivers, formal caregivers and care managers)  Informal caregiver, partly proxy by formal caregivers and care managers  Nursing staff | The findings highlight the importance of involving all types of potential users directly and early on. It demands various strategies to mitigate potential problems.    Not the technology itself causes ethical problems, but the way in which they could be used | Explorative qualitative study |
| Brodersen, 2015  Denmark  *The Smart Foor: How a public-private partnership co-developed a heterogeneous healthcare technology system* | Nursing home (n=5) in Denmark/  In partnership focus on representatives from floor company, interface designers, technology consultants, lead-users. Healthcare staff, municipality | Nursing home for more than 100 residents  Smart floor was built into nursing home during construction in 2013.  Data collection 8 months start sept. 2014 | The Smart Floor,  A smart floor registers a person's position in the room by measuring pressure and fluid. To individualize and preventive assistance, preventing fall accidents, and increasing privacy for elderly residents | To illustrate how the heterogeneous technological system (The Smart Floor) was co-developed and redesigned during knowledge sharing processes with companies, lead-users and healthcare staff, and to discuss how care practices have changed as a result of The Smart Floor system | Residents (proxy  from nursing staff)  Informal caregivers= relatives (Proxy from nursing staff)  Nursing staff (proxy from head of nursing home)  Developers  Management  Municipal leaders= LTC Administrators | Clients/ proxy: no unnecessary interruptions of daily life  Family caregiver: concerns were minimal/ instead positive reactions were seen  Nursing staff: were open to introduction of technologies/  Implementing was co-development  Management: technologies do not lead to large reduction of healthcare staff  Weak scripts of software challenged domestication process, The continuous co-development and redesign is an intensive process | Qualitative research, ethnographic |
| Coahran et. al.  2018  Canada  *Automated fall detection technology in inpatient geriatric psychiatry: nurses perceptions and lessons learned* | Mental health hospital (n=1) in Ontario/  geriatric psychiatry units (n=2), 55 beds  Unit 1: psychological and behavioural symptoms by in cognitive impaired pt,  unit 2: variety of psychiatric illnesses | 6 patients consented with Helper system (100% diagnosis dementia)  9 nurses were interviewed  12 weeks pilot | The Helper (health evaluation logging and personal emergency response) system: a ceiling mounted fall detection system that sends an alert to a smartphone when a fall is detected | Threefold purpose:  To explore nursing staff perceptions of all detection technology and its value to clinical practice  To report on lessons learned about conducting technology evaluations in authentic environments  To describe the performance of the system in detecting falls in order to identify still-needed technological improvements | Informal caregiver (proxy from nursing staff)  Nursing staff | Formal caregiver/ 5 themes:  *-Nurses are supportive of new technology that contributes to improvements in clinical practice and patient care, particularly when it functions as intended*  *-While detection is valuable, technology should focus on fall prevention*  -*Fall-alerting mechanism should be easy to use*   -*The Helper system has positive features*  -*Technological issues with the system and infrastructure limit current usefulness in practice* | Mixed methods  Quantitative (pilot) and qualitative (interviews) research |
| Dorsten et. al.  2009  USA  *Ethical perspectives on emerging assistive technologies: insights from focus groups with stakeholders in long-term care* | LTC facilities (n=7) in Pennsylvania | Residents, family caregivers direct caregivers, staff, maintenance staff, administrators from a spectrum of settings that included independent apartments, assisted living, skilled nursing, and a specialized dementia care unit    18 focus groups with semi-structured interview guides and one interview | An overview of assistive technologies (AT) were mentioned: wearable devices e.g. wristband (monitor location), environmental sensors to detect motion, lighting or noise, and integrated audio and video sensor sets to detect falls, changes in gait or changes in behaviour | This study addresses some of the gaps in our understanding of the ethical implications of technology implementation and its user acceptance in LTC facilities since AT are emerging at a rapid rate and will be increasingly utilized to compensate for tremendous staffing shortages in these facilities | LTC residents,  Family caregivers,  (proxy from nursing staff)  Nursing staff (licenced or registered nurses and certified nursing assistants)  Rehabilitation  Specialists (physicians)  Facility maintenance staff members  LTC administrators | Overall:  Concerns for privacy, autonomy, cost, safety associated with implementation of novel technologies  The relative importance of each theme varied by stakeholder group | Qualitative research design / explorative  with focus group interviews, originating out of grounded theory methodology |
| Dugstad et. al.  2019  Norway  *Towards successful digital transformation through co-creation: a longitudinal study of a four-year implementation of digital monitoring technology in residential care for persons with dementia* | Municipal healthcare organizations (n=8), dementia care ward in nursing homes  Care providers were the main group of users to adopt the technology | 172 participants from nursing staff, ICT staff, vendors, researchers, non-governmental organisations, other public organisations, innovation and funding agencies, external experts.  21 interviews, 2focus group interviews, workshops, observations training sessions, meetings | The IAT (intelligent assistive technology) system allowed integration of e.g. bed-exit or door sensors from different manufacturers.  67 installations of monitoring technology in the eight nursing homes | This study aimed  to identify the facilitators and barriers for implementation of digital monitoring technology in residential care for persons with dementia and wandering behaviour, | Resident (proxy from formal caregivers and IT)  Informal caregiver (proxy from formal caregivers)  Nursing staff  Manager  Project manager  IT staff  Macro level/ LTC administrators  Vendors  Maintenance: Janitors/ cleaning staff/ substitute personnel | Applying surveillance technologies requires a multi stakeholder perspective/ preparedness for co-creation  Outcomes from resident (proxy), informal caregiver, professional caregivers, managers, project manager, IT, vendors, janitors/ cleaning staff | Qualitative  Longitudinal case study (4 years) with elements of transformational action research / |
| Emilsson (2023)  Sweden  *Experiences of using surveillance cameras as a monitoring solution at nursing homes: The eldercare personnel’s perspective* | Nursing homes (n=3) in Sweden. About 50 residents (majority cognitive impaired) of which 44 of them had cameras during the study | 16 participants:  12 Staff members who worked night shifts  3 daytime working staff members in order to catch effects that emerged during the day  1 unit manager | Surveillance cameras (SC) | To analyse obstacles and opportunities associated with implementation/ use of SC at nursing homes, from perspectives of eldercare personnel and conditions of the older people with cognitive impairment who live in nursing homes | Nursing staff | Prerequisites: Need for adequate information and education at the right moment of implementation. Need for sufficient support also during nighttime, when most used.  Maintain integrity for residents, family caregivers and professional caregivers.  Sufficient infrastructure to maintain security for residents | Qualitative research design |
| Engström et.al.  (2005)  Sweden  Staff perceptions of job satisfaction and life situation before and 6 and 12 months after increased information technology support in dementia care | Residential home (n=1) for persons with dementia.  4 of the 6 units were selected with 9-12 residents in each,  Units connected by an indoor walk | Participants: 33 staff members  Baseline, 6 and 12 months after installing new technology, within and between-groups comparison  Experimental group: staff from two units,  Control group: staff from two other units of the same residential home | A passage alarm activated through sensors that residents wore around their necks or in a pocket), passage alarm in the garden (pressure sensors on the fence) and fall detectors and movement detectors near the bed (pressure mats).  Sensor-activated illumination of the toilet | To measure staff members' satisfaction with their work before and after increased information technology (In support in dementia care. Comparisons were also performed of perceived life satisfaction and sense of coherence | Nursing staff | Staff members' perception of psychosocial job satisfaction and quality of care aspects improved in the experimental group in comparison with the control group. The factors personal development, workload, expectations and demands, internal motivation and documentation, as well as total scores on 'psychosocial aspects of job satisfaction' and 'quality of care aspects' increased in the experimental group. | Quantitative research  Quasi-experimental, non-equivalent groups design was used |
| Fraile et. al.  (2010)  Spain  *Applying Wearable Solutions in Dependent Environments* | Case study in geriatric care facility (n=1) | 50 residents  10 nurses  2 doctors | MAS (multi-agent system): wearable devices/ mobile technology for the care of residents in a geriatric care facility.  Based on advanced ZigBee wireless network (WSN), includes location and identification microchips installed in patient clothing or caregiver uniforms. | This paper describes the smart wearable device (MaRV) MAS, which is intended to automatically manage and improve the assistance to patients in geriatric facilities by using smart wearable technology. | Residents (Proxy from nurses and doctors)  Nursing staff  Doctors  Developers | Initial high error rate however resulys are promising, residents’ security was improved. The system still requires much work | Qualitative  case study with pilot in real life setting and survey |
| Hall et. al.  (2017)  UK  *Implementing monitoring technologies in care homes for people with dementia: A qualitative exploration using Normalization Process Theory* | Multiple case study in dementia-specialist care homes (n=3) in North-West England  Conifer gardens and Sycamore Lane= with nursing  Heather Grove= without nursing | 42 participants  (24 staff,  9 relatives,  9 residents of which 3 able to participate in interviews)  Observations  36 semi-struct. Interviews  Examination of documentation  Care records data extraction | A nurse call system (all 3 care homes)  incorporating bed-exit monitoring and staff alerting. Other monitoring technologies, e.g. wearable activity tracker (Sycamore Lane), wearable location-tracking technology (Conifer Gardens), and door monitoring (Heather Grove) | To explore facilitators and barriers to the uptake of monitoring technologies into routine practice in care homes.  To explore the influence of the ethical debate between ‘safety’ and ‘freedom’, the perception of benefits from using monitoring technologies balanced against the potential challenges | Nursing staff (registered nurses, clinical specialists, care workers)  Senior managers  Family caregivers (relatives)  Residents | Grouped in themes:  -Reasons for application, most common reason is to enhance safety, to have staff where they were most needed at the right time  -Ways MT were implemented, training is valuable, sometimes lack of trust, use MT to monitor staff  -Use of MT in practice, unknown with technology/ generating alarms, damages, overburden. Not all collected information is useful | Qualitative study,  Embedded multiple-case study with qualitative methods |
| Hall et.al.  (2019)  UK  *Moving beyond ‘safety’ versus ‘autonomy’: a qualitative exploration of the ethics of using monitoring technologies in long-term dementia care* | Multiple case study in dementia specialist care homes (n=3) in North-West England  Conifer gardens and Sycamore Lane= with nursing  Heather Grove= without nursing | 42 participants  (24 staff, 9 relatives, 9 residents of which 3 able to participate in interviews, 9 resident care records were data extracted)  Observations  36 semi-struct. Interviews  Examination of documentation  Care records data extraction | A nurse call system (all 3 care homes)  incorporating bed-exit monitoring and staff alerting. Other monitoring technologies, e.g. wearable activity tracker (Sycamore Lane), wearable location-tracking technology (Conifer Gardens), and door monitoring (Heather Grove) | To explore the extent to which remote monitoring of care home staff, and equality of access to technologies, influenced the use of monitoring technologies within the routine practice, and to discuss subsequent ethical implications | Nursing staff (registered nurses, clinical specialists and care workers)  Senior managers  Relatives  Residents | Five themes:  Understanding  Business/ environmental influences  Reasons for using technologies  Ways of implementation  Use of technologies in practice  MT: used to enhance safety, to monitor workforce  Data of MT can be used to defend against accusations from family however data of MT (without camera) are no hard evidence of delivered care | Qualitative research  An embedded multiple-case study design was used |
| Holmes et. al.  (2007)  USA, New York  *An evaluation of a monitoring system intervention* | Large nursing home (n=1), two special care units (SCU) for people with cognitive impairment  Comparison of use of Vigil, a monitoring system in one SCU and one unit without Vigil | 92 residents, 66 completed all waves of data collection | Vigil monitoring system:  A bed exit sensor positioned under each resident's bed sheet. Bathroom and bedroom exit monitors  An incontinence sensor is also available (in this study not used) | To assess the extent to which modern technology can augment or substitute for direct staff intervention in non-acute late-evening and nighttime situations in the nursing home setting | Residents (proxy/ written about from quantitative data)  Nursing staff | No reduction in falls or injuries when monitoring system is implemented  No effect in staff burden after implementing monitoring system  Relevant factors:   -Resistance of staff in implementation of monitoring system  -Nursing staff reluctant to change their methods of car | Quantitative research  Quasi-experimental design with variation of a cluster randomized trial: intervention and comparison group |
| Lauriks et.al.  (2020)  The Netherlands  *Effects of Assistive Home Technology on quality of life and falls of people with dementia and job satisfaction of caregivers: Results from a pilot randomized controlled trial* | Nursing home in Amsterdam (n=1),  Five new group homes of each 6 residents with assistive home technology (AHT)  4 new group homes of each 6 clients, without AHT | 54 people with dementia on nine care group homes  25 caregivers | AHT: ‘house-functions’ (lights, doors). AHT also contains systems that alert caregivers when the safety of a resident could be at risk.  Intervention group:  ● life circles  ● pathway lighting,  ● automated lighting,  ● automated alerts,  ● automated sun blinds | To determine the effects of AHT on outcomes in residents with dementia and staff | Residents (partly proxy by nursing staff)  Nursing staff of group homes | Overall: A trend in reduction of fall incidents during nighttime bathroom/ toilet visits in group homes with AHT  AHT did not affect use of physical restraints  Residents: Positive effect on some aspects of QoL, less social isolation, more things to do. No effect on satisfaction with care  Caregivers:  No significant effect on job satisfaction and workload  Reduced appreciation due to extra efforts and malfunctions | Quantitative research design  Pilot randomized controlled trial in nine in-patient care group homes (group homes with. and without AHT) |
| Meng et.al.  (2020)  China  *Status and Influential Factors of Intelligent Healthcare in Nursing Homes in China* | Survey was carried out/ ‘National Training Courses for Directors of Nursing homes’ | 197 administrators of nursing homes in China participated  A survey was carried out (Sept. 2017-May 2018): | Health care products, divided into categories:  medical care services  chronic disease management, condition monitoring,  Life care services (fall monitoring and wireless positioning)  Intelligent nursing devices: smart mattresses,  intelligent monitoring beds, smart wheelchairs  Portable health monitoring devices | To explore the current status of intelligent healthcare service (HIS) in nursing homes in China and its related influential factors | Nursing staff (proxy from administrators)  Nursing home administrators | 79,69% of nursing homes provided IHS  Factors related with provision of IHS: attitudes of staff, residents receptiveness to IHS.  Restrictive factors: lack of financial investment, residents low acceptance rate, lack of technical management personnel.  Factors related to effectiveness: financial investment, attitudes of staff towards IHS, proportion of residents favouring IHS  Attitudes of stakeholders play pivotal roles in provision of HIS | Quantitative Cross sectional study  A questionnaire on IHS in nursing homes |
| Niemeijer  (2010)  The Netherlands  *The ideal application of surveillance technology in residential acre for people with dementia* | Concept mapping with professional caregivers  and academics | Professional caregivers (n=9) and academics (n=6)  Total group:  Two elderly care physicians  Two psychologists  Two ethicists  Three registered nurses  Six certified nurse assistants | Surveillance technology (ST)  definition Niemeijer, 2015: tagging and tracking technology, sensors, audio-and video surveillance | To explore how ST is viewed by care professionals and ethicists working in the field by investigating the ideal application of ST in the residential care for people with dementia | Nursing staff  Academics | Professional caregivers rate safety of residents higher than the experience of freedom, academics the other way around.  Training and understanding of ST is necessary,  The more responsibility the caregiver has for the resident, the more safety become a an issue in relation to the experience of freedom | Qualitative research |
| Niemeijer  (2014)  NL  *The Use of Surveillance Technology in Residential Facilities for People with Dementia or Intellectual Disabilities: A Study Among Nurses and Support Staff* | Field observation in nursing home (n=1), informal conversations and 8 formal interviews | 1 nursing home 22 nursing staff,  1 care facility for people with ID  16 support workers | Surveillance technology (ST) DECT phones to ‘listen on’  Motion sensor:  Acoustic sensors and surveillance  Electronic bracelets (with programmed automatic doors, ‘living circles’  Automatic doors with access code  GPS tags (in clothing)  Video surveillance | To investigate how ST is being used by nurses and support staff in LTC facilities for people with dementia or intellectual disabilities, in order to explore possible benefits and drawbacks of ST in practice and to explore residents experiences and use of ST, in order to assess whether and how ST might increase the resident’s autonomy | Nursing staff  Management  Elder care physician | Professional caregivers kept continuing doing rounds at night due to various reasons  Alarm fatigue due to false-alarms  Keeping clients in close proximity due to not fully functioning GPS/ electronic bracelet  Locking doors for protection against what could happen  Forget to take devices of when not necessary anymore/ evaluation not in time | Qualitative ethnographic field study |
| Niemeijer  (2015)  NL  *The experiences of people with dementia and intellectual disabilities with surveillance technologies in residential care* | Participant observation with informal conversations with residents  8 interviews with relevant others (family and staff)  Care facility for people with intellectual disabilities (ID)  42 clients, 7 residents per small-scale unit | Nursing home 43 residents (6 small-scale units each 6 clients, one large-scale unit 13 clients) | Surveillance technology (ST) DECT phones to ‘listen on’  Motion sensor:  Acoustic sensors and surveillance  Electronic bracelets (with programmed automatic doors, ‘living circles’  Automatic doors with access code  GPS tags (in clothing)  Video surveillance | To explore how residents in residential care experienced ST, in order to assess how ST might influence the autonomy of people with dementia and ID | Residents (partly proxy by nurses)  Nursing staff | Theme 1 Wandering around:  less restless in night due to more walking during day  Getting lost: inconvenience by more freedom, feeling lost/ unsafe/ slipping through doors  Being triggered: agitation as other clients go through doors  Retreating to new spaces: carving out extra private space  Theme 2: Feeling stigmatized, ‘like a patient’. Missing the company to walk with resulting in less walks: used to  Not wanted to be watched | Qualitative ethnographic field study |
| Nijhof et.al. (2012)  NL  *How assistive technology can support dementia care: A study about the effects of the IST Vivago watch on patients’ sleeping behavior and the care delivery process in a nursing home* | Nursing home (n=1) | 7 residents wore the Vivago watch  5 caregivers were interviewed  3 months period  Data collection:  diary, observations, interviews and monitoring data | Monitoring technology; the Vivago Watch, measures the sleeptime, sleep periods, circadian rhythm and the sleep/wake rhythm. An accelerometer is embedded which measures the acceleration of the wrist | The main purpose of this study was to gain insights into the effects of the watch on the sleep/wake rhythm and on the care delivery process of patients with dementia. | Proxy:  Residents (proxy by caregivers)  Nursing staff | Residents/ proxy from caregivers: Acceptability is an important issue, watch can be improved (too big, hard strip)  Caregivers: Introduction took too much time/ not everyone informed, re-organisation at same time is not ideal  Caregivers were sceptical about the watch before using it  Opinion leaders are important  Usability: Appearance needs to be improved, strip irritates skin  Infrastructure can be improved to be more supportive | Mixed-methods design, qualitative and quantitative |
| Offermann-van Heek et.al. (2018)  Germany  *They Don’t Care About Us! Care Personnel’s Perspectives on Ambient Assisted Living Technology Usage: Scenario-Based Survey Study* | Care setting of professionals: medical care, geriatric care, disabled people’s care (DPC) | E-survey (172 participants April -June 2017): distributed in web based networks of geriatric care and nursing care  Interviews with 6 professional caregivers | 12 different ambient assisted living (AAL) systems: Emergency button caregiver and caretaker,  Fall sensors (floor / body), Room sensors (air, temperature)  Motion detectors in rooms, Motion sensors in shoes,  Infrared cameras  Microphones  Video cameras | To investigate professional caregivers’ AAL technology acceptance and their perception regarding specific technologies, data handling, perceived benefits, and barriers.  To investigate whether the different care contexts influenced the acceptance of assistive technologies | Nursing staff | Technology perception:  Participants working in DPC indicated a lower acceptance and more negative attitude of AAL technologies than those working in geriatric and medical care es care (DPC)  Specific differences in data, technology and acceptance evaluations | Mixed methods  First part: Qualitative research design (interviews)  Second part: quantitative research design |
| Sallinen et.al.  (2015)  Finland  *Technology and active agency of older adults living in service house environment* | Service houses (n=3) in Western Finland  Thematic interviews of older residents | 12 residents of service houses in Western Finland  Residents with cognitive problems were not excluded | Low tech or midtech technologies e.g. alarm wristbands, safety floors, various communication and monitoring systems or assistive devices for mobility, hearing or vision | To explore whether or not the currently used technology supports the agency and independence of the residents | Residents (partly proxy from informal caregiver, 3 of 12 residents) | Results are presented in the six modalities of agency  Knowing how to do something  Being able to do something  Having to do something  Having the possibility to do something  Wanting to do something  Appreciating something | Qualitative research design |
| Sallinen et.al.  (2019)  Finland  *Ethical dilemmas related to the use of safety technology in service house environments* | Service house units (n=2) in South-West Finland | 12 residents  5 relatives of two service house units in South-West Finland  Older adults with memory problems were encouraged to participate  (3 of 12 cases family was needed in conversation  Thematic interviews | Technologies representing systems to detect fall risks or hazards, or can be used to alarm when assistance is needed. Examples: safety floors, various monitoring and alarm systems, smoke and temperature detectors | To explore residents’ and informal caregivers’ ethical dilemmas concerning safety technology (ST) in service houses. To enhance the active participation of the residents / encouraging them to express their worries, hopes, need, expectations toward ST | Residents (partly proxy from informal caregiver, 3 of 12)  Informal caregivers (Relatives) | Answers from residents and relatives on three themes:  Supervision versus privacy  Fear of losing human contact  Autonomy and freedom | Qualitative explorative research |
| Soto-Mendoza et.al.  (2015)  Mexico  *Design of a Predictive Scheduling System to Improve Assisted Living Services for Elders* | Geriatric centres: 19 Interviews, 180h observations to understand the nursing environment  To develop and test and evaluate prototypes | 11 Caregivers and 12 residents in geriatric centres | PRESENCE triggers real-time alerts of risky situations (falls, entering off-limits areas and informs caregivers of routine tasks that need to be performed | To explore and validate the main characteristics of the predictive schedule, including providing notifications about risky situations  To develop suitable assistive applications based on the principal characteristics found in naturalistic environments. | Nursing staff  Residents (proxy from nursing staff/ researchers)  Developers | For residents this application was less useful  Caregivers: predictive schedule could be helpful. Following a strict sequence is complex.  Alerting notifications were not adequate/ annoying  Developers: Activity recording should facilitate and minimize the workload of caregivers/ PRESENCE/ SSAMI was not adequate for this purpose | Qualitative explorative research |
| Stark et.al.  (2017)  Switzerland  *Effects of a mobility monitoring system on the cost of care in relation to reimbursement to Swiss nursing homes: learnings from a RCT* | RCT in nursing homes (=3) in Switzerland, 11 wards, 44 clients  During 10 weeks  Intervention group: use of monitoring system  Control group | 44 Residents with cognitive impairment and sleeping disorders | An in-bed system that records mobility and micro-activity without any body contact, a bed-exit alarm can be activated. | To investigate whether a mobile monitoring system accompanied with case conferences would improve sleep quality in nursing homes and to investigate the influence of this monitoring system on the costs of care | Nursing staff  Management | It is not shown that the use of a monitoring system reduces the activities of nurses, as well no differences in costs were seen  In intervention group care activities in nighttime increased in phase 2 after a decrease in first phase. A growing need of care is seen over time | Quantitative research, two-phase randomized controlled trial |
| Te Boekhorst  (2012)  The Netherlands  *Quality of life of nursing-home residents with dementia subject to surveillance technology versus physical restraints: an explorative study* | Nursing homes (n=6) in NL  Two groups:  With one or more forms of surveillance technology  With one of more forms of physical restraints | 221 residents (150 took part in all three measurements) | Acoustic and visual monitoring systems, door sensors and infrared sensors (beds or chairs) that detect movement and send an alarm to nursing staff, chips sown into residents’ clothing or shoes that are programmed to close or open doors, or use of the Global Positioning System (GPS) to assess resident’s location | To explore whether nursing-home residents with dementia subjected to surveillance technology had better quality of life scores for mood, behavioural and societal dimensions than residents with physical restraints | Residents (proxy by nursing staff as they answered questionnaire) | More physical restraint use for residents with higher ADL dependency  Surveillance technology is more often used for people with medium ADL dependency and less advanced stages of dementia  There was no significant effect of surveillance technology on the affect of residents  The introduction of surveillance technology does not have a significant effect on the use of physical restraints | Quantitative research  Non-experimental longitudinal research design |
| Wigg  (2010)  USA  *Liberating the wanderers: using technology to unlock the doors for those living with dementia* | Nursing homes (n=2) in USA,  Pine Tree Place, with 30 residents and locked doors and Oceanside Vista, small scale facility 8 residents | 60 residents with dementia in two nursing homes  Observations of residents in both nursing homes and informal interviews with staff.  Oceanside Vista, 10 years observation  Pine Tree place: 7 months observation | Motion detection, locked doors, a walking loop around the perimeter of the living and dining space. | To examine the impact of locked doors on residents with dementia and to compare their experiences with those of residents with dementia living in an unlocked environment | Residents (partly proxy based on observations author and interviews with nursing staff) | Similar to the thwarted desire to go home, residents’ inability to go outdoors increased their anxiety. The ability to move and exercise often lessened the anxiety levels. The need for pharmacological intervention of anxiety was often reduced  The ability to leave and enter the outdoors recognizes the potential human need to move, not being locked behind doors, not struggling to open a locked door | Qualitative explorative research |
| Zwijsen et.al.  2012  Nr. 35, pdf 95  The Netherlands  *Surveillance technology: An alternative to physical restraints? A qualitative study among rofessionals working in nursing homes for people with dementia* | Key persons from nursing homes (n=7) | Semi-structured interviews with 9 key persons from 7 nursing homes  8 focus groups with 4-8 participants (nurses and multidisciplinary teams) | Acoustic monitoring, chips worn in clothing, inactivity sensors, movement sensors, door sensors, bed pressure sensors | To obtain an insight into the view of Dutch dementia care professionals on the feasibility of surveillance technology as an alternative to physical restraints | Nursing staff  Managers  Physicians | Main goal of using ST is providing safety and ‘a peace of mind’ nurses and relatives  ST: provides additional safety, not harmful for the resident,  ST can increase freedom/ less need to disturb the resident  ST cannot prevent falls, cannot guarantee quick help/ not always is help indeed needed  ST devices do not always work properly/ fragile/ tend to do extra rounds to check  ST could violate privacy | Qualitative research  With semi-structured interviews and focus groups |
